# Laminectomy in Fracture of the Spine

**Published:** 1901-11-30

**Authors:** 


### LAMINECTOMY IN FRACTURE OF THE SPINE.

Laminectomy must often, at first sight, appear so reasonable and hopeful a proceeding when symptoms of pressure upon the spinal cord are associated with those of fracture of the spine, that it is well to look at the other side of the question lest we should be led by surgical enthusiasm into adventurous but useless operative measures. In a clinical lecture delivered at St. George's Hospital, Mr. Clinton Dent [1] points out the hopelessness of laminectomy in any such cases, even though one succeeds in reaching definitely the bone which is pressing on the cord. For the mischief has been done, and the destruction that has already been produced is often so great that recovery is out of the question. The case is not analogous with one of trephining of the skull. In the brain there is, so to speak, brain tissue to spare, but that is not the case with regard to the spinal cord. Then it by no means follows that the main injury to the cord will be at the same level as the fracture of the bone. Very seldom indeed will it happen that a portion of the lamina has been driven right into the spinal cord. In such a case it would, no doubt, be right to remove or raise up the offending piece of bone; but even then, although the mechanical condition be completely removed, the benefit will probably be far less than in a case of compressed or wounded brain. Myelitis may readily supervene, and in any event the recovery of the cord to a sufficient degree to discharge usefully its functions would be highly improbable. In a case where a severe blow had been received on the back from a sharp-edged instrument, it might be proper to cut down on the part in the hope of doing some good. But in a diffused injury, such as results from a fall from a height, it is almost certain that the fracture will be found in the bodies of the vertebræ, and that if the lamina is broken at all, the fragments will not be found to compress the cord. Mr. Clinton Dent said that he saw many cases in South Africa of gunshot wounds of the spine, which were frequent, because the wounds were so often received while the men were lying down facing the enemy. It had been hoped that surgery might do much for these injuries, but such hopes had been altogether disappointed. He related details of a case which showed that the passage of a high velocity bullet through the spine might, even without opening the theca, produce such tremendous vibration effects as to cause hopeless injury to the nerve tissue. In this case "the cord was not directly injured by the bullet, which had merely passed close to it." But it was pulpified all the same, and "if we had taken away every scrap of bone that might have compressed the cord in that soldier, the operation would have done no good." Brilliant as have been the results of laminectomy in some cases of new growth, the huge majority of traumatic cases seem to be as far as ever removed from the domain of practical operative surgery.

[1] Clinical Jour., Nov. 20.

